# A systematic review of pathophysiology and management of familial hyperaldosteronism type 1 in pregnancy

**DOI:** 10.1007/s12020-021-02763-5

**Published:** 2021-05-27

**Authors:** Viola Sanga, Teresa Maria Seccia, Gian Paolo Rossi

**Affiliations:** 1 Hypertension and Emergency Unit, Department of Medicine - DIMED, University of Padua, Padova, Italy; 2 PhD Arterial Hypertension and Vascular Biology, Department of Medicine – DIMED, University of Padua, Padova, Italy

**Keywords:** Hypertension, Pregnancy, Primary Hyperaldosteronism, Familial Hyperaldosteronism type 1, Glucocorticoid-remediable aldosteronism

## Abstract

**Purpose:**

Familial hyperaldosteronism type 1 (FH-1) is a rare autosomal dominant form of primary aldosteronism, which features a marked phenotypic heterogeneity, ranging from mild to severe forms of arterial hypertension that can be complicated by stroke and cardiovascular events at a young age. As affected patients usually reach the fertile age, transmission of the disease to offspring is common. Notwithstanding this, reports of FH-1 in pregnancy are limited and there is a lack of treatment guidelines.

**Methods and results:**

We searched the PubMed and EuropePMC databases with a PICO strategy to retrieve available information on management of FH-1 patients during pregnancy. We could identify seven relevant articles, which are herein reviewed.

**Conclusion:**

Based on available information on pathophysiology and treatment of FH-1 in pregnancy, recommendations for the rational management of FH-1 in pregnancy are provided.

## Introduction

In 1969, a familial form of primary aldosteronism (PA) relieved by dexamethasone treatment was reported and defined glucocorticoid-remediable-aldosteronism (GRA) (OMIM: 103900) [1, 2]. As additional forms of familial hyperaldosteronism (FH) were thereafter identified [3–6], GRA was thereafter designated as FH type 1 (FH-1) [7].

FH-1 is transmitted as an autosomal dominant disorder and is characterized by a marked phenotypic heterogeneity. Reported clinical phenotypes ranged from mild arterial hypertension and aldosteronism to severe forms complicated by cardiovascular events, mainly ischemic or hemorrhagic strokes, occurring at a young age [8, 9].

In 1992, Lifton’s laboratory identified a chimeric gene [10] resulting from homologous recombination between the genes encoding 11-β hydroxylase (CYP11B1) and aldosterone synthase (CYP11B2). These genes share 95% homology and map close to each other on chromosome 8q24. In the chimeric gene, acquisition of the adrenocorticotropic hormone (ACTH) responsive elements of CYP11B1, along with the CYP11B2 sequences encoding aldosterone synthase, explained both the ectopic expression of aldosterone synthase in the adrenocortical zona fasciculata, which normally secretes cortisol, and the correction of FH-1, i.e., resolution of the hyperaldosteronism and normalization of the high blood pressure (BP) values, with suppression of the ACTH-drive by glucocorticoid treatment.

This discovery lead to develop genetic testing based on long polymerase chain reaction (PCR) [11], which permits a conclusive diagnosis of FH-1, and therefore, institution of glucocorticoid treatment. FH-1 is rare in that it affects <1% of PA patients [12, 13]. However, since PA is common in hypertensive patients [14, 15], but often overlooked, the prevalence rate of FH-1, particularly of its milder forms, likely is markedly underestimated, as most FH-1 patients present with mild clinical PA phenotypes. Therefore, FH-1 patients usually reach the fertile age, which allows transmission of the disease to offspring. However, pedigrees entailing fatal strokes and aortic rupture occurring at a young age have also been reported [9]. In spite of this, information on pathophysiology and management of FH-1 in pregnancy is scant [16–19] and treatment guidelines lack. Hence, we have searched in the literature for available information on pathophysiology and course of FH-1 in pregnancy, with the final goal of providing some recommendations for the management of this condition.

## Methods

We searched the PubMed and EuropePMC databases with a PICO strategy (Table S1) to identify available information/papers on the best management of FH-1 patients during pregnancy.

The PRISMA strategy was then applied using the following boolean operators: [‘Familial hyperaldosteronism type 1’ OR ‘Glucocorticoid remediable aldosteronism’] AND [‘pregnant’ OR ‘pregnancy’] (Fig. S1).

## Results

In total, 400 papers containing the searched terms (PRISMA graph Fig. S1) were found, but only seven comprising anecdotal cases were judged to be relevant for the management of FH-1 patients during pregnancy (Table 1 and Table S2), indicating that systematic research efforts are needed on this topic.

**Table 1:** Blood pressure, treatment and complications in published cases of FH-1 in pregnancy

Reference	Year	N. of patients	N. of families	N. of pregn.	Maternal age	BP and related complications	Antihypertensive therapy during pregnancy	Other complication during pregnancy/delivery	Rate of cesarean sections and indications
Wyckoff et al. [18]	1999	16	Not specified	35	25 ± 6	74% HT–66% chronic HT (39% had PAH, 17% had resolution of HT) 6% preeclampsia 3% transient HT	23% required ≥1 anti-hypertensive medications: methyldopa (*n* = 2) potassium-sparing diuretics (*n* = 3) beta-blockers (*n* = 2) thiazides (*n* = 5)	6% chorioamnionitis 6% failure of placental separation 12% >500 cc blood loss during delivery 0% eclampsia or HELLP syndrome	43% (32% primary) (A) Indications for primary cesarean section: fetal distress (*n* = 4); failure of labor to progress (*n* = 3); severe HT (*n* = 2); breech presentation (*n* = 2); fever (*n* = 2); partial placenta previa (*n* = 2); prolapsed cord (*n* = 1); nuchal cord (*n* = 1); twin pregnancy (*n* = 1); head entrapment (*n* = 1). (B) Indications for secondary cesarean section: repeat cesarean section (*n* = 5); twin pregnancy (*n* = 1); fetal distress (*n* = 1).
Mulatero et al. [19]	2002	8	1	29	Not specified	(in 17 over 29 pregn.) 0% PAH, preeclampsia or transient HT 6% chronic HT	Not specified	None	None
Hamilton et al. [17]	2009	1	1	1	28	Normal BP during gestation without therapy; then, BP increase at 1 month postpartum	None (discontinued verapamil once pregnancy was known)	None	None
Campino et al. [16]	2015	1	1	1	21	Normal BP during gestation without therapy; then, BP increase in the postpartum period	None (discontinued dexa. 0.25 mg once pregnancy was known)	None	None
Sanga et al. [20]	2020	1	1	1	31	BP normalization during 1st trimester with no therapy; BP increase at the end of 2nd trimester	Discontinued dexa. 0.50 mg once pregnancy was known; restarted dexa. 0.25 mg o.d. at the end of 2nd trimester.	None	None

*Pregn.* pregnancies, *PAH* pregnancy-aggravated hypertension, *HT* hypertension, *BP* blood pressure, *dexa*. dexamethasone

In 1999 a retrospective study of the maternal and fetal outcomes of 35 pregnancies in 16 women with proven FH-1, albeit of undetermined severity, concluded that in FH-1 women there was no evidence for an excess incidence of preeclampsia, which occurred at a rate (6%) well within the range (between 2.5 and 10%) seen in the general obstetric population [18]. No cases of overt eclampsia, or the syndrome of hemolysis, elevated liver function tests, and low platelets (HELLP) were seen. In hypertensive women an exacerbation of hypertension during gestation occurred in 39% of the pregnancies and resolution of hypertension was reported in 17% of the pregnancies. It remained uncertain when resolution occurred, because the study did not report the time course of hypertension. Of note, 23% of the pregnancies (*n* = 8) required one or more antihypertensive medications; all these women were reported to have hypertension before pregnancy and 17% were on antihypertensive medications from the onset of pregnancy. A cesarean section was performed in 43% of the pregnancies. A trend toward a lower weight at birth was seen in the infants of the FH-1 mothers who experienced pregnancy-aggravated hypertension.

In 2002, 29 pregnancies in eight FH-1 women were reported in a five-generation FH-1 benign pedigree with a mild phenotype entailing a high number of normotensive or mildly hypertensive members and a much lower frequency of stroke than in other FH-1 pedigrees [19]. No pregnancies were complicated by preeclampsia and no cesarean sections were performed, indicating an uneventful course of pregnancy in this pedigree.

In the following decades only two other cases of FH-1 in pregnancy were reported [16, 17]. Hamilton et al. [17] described a woman from a pedigree with young onset hypertension and stroke, who presented at age 28 years with high BP (160/90 mmHg). The woman and her father tested positive for FH-1. She was treated with the non-dihydropiridine calcium channel blocker verapamil, after which she became pregnant and normotensive and verapamil could be withdrawn. Notwithstanding this, her BP values remained normal without medications throughout pregnancy. Her plasma renin activity (PRA) progressively rose to the upper normal range of 2.7 ng/ml/h at week 18th and to elevated values (7.0 ng/ml/h) at week 24th of gestation, while plasma aldosterone concentration (PAC) values fell within the normal range (260 pmol/L equivalent to 9.4 ng/dl) at week 18th, and then became elevated (685 pmol/L equivalent to 24.7 ng/dl) at week 24th. Information on delivery and/or newborn outcome were missing. At 1-month postpartum, the BP values raised to 160/120 mmHg; PRA was suppressed (0.29 ng/ml/h) and PAC remained overtly high (605 pmol/L equivalent to 21.8 ng/dl), resulting in an unambiguously elevated ARR of 90 ng/mIU (n.v. < 20.6 ng/mIU). Prednisolone treatment was commenced at 2.5 mg daily, but whether BP values normalized with such treatment was not reported.

In 2014, Campino et al. [16] described a 21 years-old FH-1 pregnant woman from a FH-1 family. At age 16, her ARR was high (70 ng/mIU); after 3 months of dexamethasone treatment (0.25 mg o.d.) both high BP and ARR values were normalized. At age 21 she became pregnant and discontinued the drug. Her BP was normal throughout gestation with no antihypertensive medications, with values ranging between 103/69 and 110/70 mmHg. However, at week 28th her ARR was borderline elevated (20 ng/mIU); at week 36th it increased to a value, which was consistent with PA (30 ng/mIU). The ARR rose further to 240 ng/mIU in the postpartum period, a rise that was accompanied by only a modest increase of BP to an average of 136/94 mmHg. Further information on treatment, BP and ARR values was missing.

We recently reported on a 31 years-old Caucasian pregnant FH-1 woman from a severe FH-1 pedigree [20] featuring a very high incidence of stroke and premature aortic rupture. At the time of presentation, her ARR, as calculated with the ARR-App [21], was more than 10-fold higher than the upper normal range (290 ng/mIU; n.v. < 20.6). After confirming the diagnosis of FH-1 by genetic testing, control of arterial hypertension and hyperaldosteronism was accomplished with low-dose dexamethasone (0.50 mg o.d. in the evening) treatment. Four years after presentation she became pregnant and was advised to stop dexamethasone treatment and to undertake close monitoring of BP and serum K^+^ values. At the end of the 2nd trimester her hypertension recurred and she was restarted on dexamethasone (0.25 mg o.d. in the evening), which rapidly normalized her high BP values. She delivered a healthy normal weight male baby at term with no complications whatsoever. The postnatal course was uneventful and the newborn growth was normal. After delivery, she restarted dexamethasone (0.50 mg o.d.) with persistent normalization of BP and PAC values. She refused to provide the baby’s DNA for a free genetic test for FH-1 notwithstanding multiple invitations. Five years later she informed us that her 5-years old child had developed severe drug-resistant hypertension, but did not accept an invitation for a free consultation.

## Discussion

Several pathophysiological considerations need to be made concerning FH-1 in pregnancy. During normal pregnancy, progesterone and aldosterone increase in parallel and this rise is held to be key for the normal development of the placenta [22]. Nonetheless, aldosterone secretion remains tightly regulated in pregnancy, which allows the mother to cope with changes of salt intake, and to develop volume expansion during pregnancy [23].

ACTH and cortisol levels also increase during pregnancy secondary to placental production of corticotropin releasing hormone (CRH) [24]. Given that that aldosterone production is ACTH-dependent in FH-1, it was, therefore, hypothesized that the ACTH increase could exacerbate hyperaldosteronism and hypertension during FH-1 pregnancy [23]. However, since progesterone acts as a mineralocorticoid receptor antagonist, its raised levels in pregnancy may block the effects of the increased aldosterone [25].

Furthermore, progesterone, but not estradiol, was reported to inhibit the chimeric isoforms of aldosterone synthase in HEK-293 cells transiently transfected with vectors containing the full chimeric CYP11B2 cDNAs [26], thus suggesting a peculiar beneficial action of this hormone in FH-1 pregnancy. Collectively, the available results point to the increase of progesterone as a plausible mechanism of BP and PAC normalization during the first trimester of pregnancy and throughout pregnancy, as noted in the two aforementioned clinical cases [16, 17], where pharmacological therapy was not necessary during pregnancy, and also up to the third trimester in the most recent case [20].

As regards treatment of FH-1 women who become aware of being pregnant during the 1st trimester, immediate withdrawal of dexamethasone with careful monitoring of BP values and serum K^+^ levels is advised, because of its potential negative effects on fetal development. However, it is worth noting that dexamethasone is the most potent and long-acting glucocorticoid drug in suppressing ACTH [27]. It was also contented that prednisolone and hydrocortisone, being inactivated by placental 11-β-hydroxysteroid dehydrogenase type 2 (11-β-HSD-2) more effectively than dexamethasone, should be preferred during pregnancy. However, since treatment of FH-1 during pregnancy is for the mother and not for the fetus, it seems logical to select the most potent ACTH-suppressive treatment with dexamethasone and prefer the lowest dose that warrants normotension and control of hypokalemia in case of BP values increase and serum K^+^ levels fall during the 3rd trimester. Exploitation of such strategy in the last case observed [20], where the effectiveness of low-dose dexamethasone was well-documented before pregnancy, warranted not only an uneventful course of gestation, but also delivery of a healthy baby, albeit likely also affected by FH-1.

**Fig. 1:**
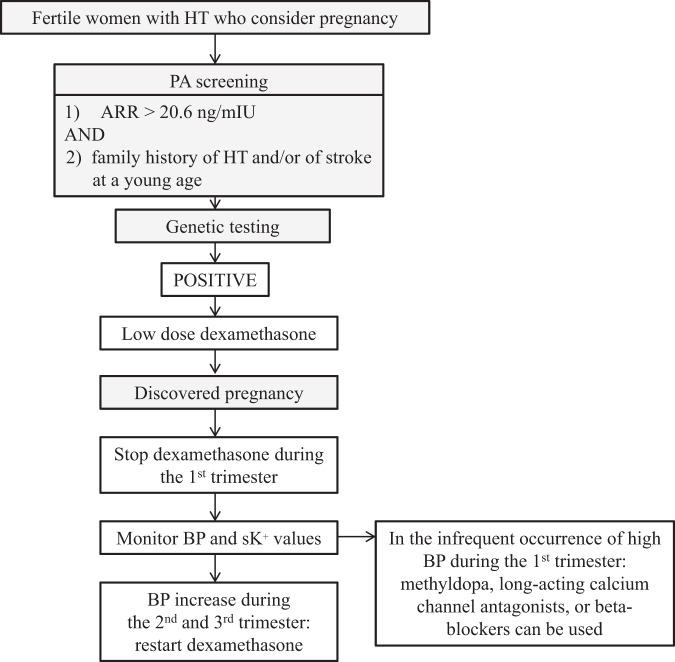
Diagnostic and therapeutic algorithm for screening and management of women with hypertension and possible FH-1. ARR aldosterone-renin ratio, HT hypertension PA primary aldosteronism

In conclusion, based on the available information in the literature, the following recommendations for the management of FH-1 women in pregnancy can be put forward. All fertile young women with arterial hypertension who consider pregnancy, should be screened beforehand for PA as indicated (Fig. 1) [28]. A raised ARR should be followed by a search for the chimeric gene in all women who have a strong family history of hypertension and/or stroke at young age. In FH-1 women who become aware of being pregnant, immediate withdrawal of dexamethasone followed by careful monitoring of BP values and serum K^+^ levels is advised during the 1st trimester. Should blood pressure become uncontrolled during this trimester, the drugs that are usually prescribed in hypertensive women during pregnancy including methyldopa, long-acting calcium channel antagonists, or beta-blockers [29], can be used. If BP rises during the 2nd and/or the 3rd trimester, low to very low dose dexamethasone is the treatment of choice with the aim of normalizing BP but also PAC, renin and serum K^+^ levels.

## Supplementary information

Supplementary Information
