# Supplementary material for: A systematic review of pathophysiology and management of familial hyperaldosteronism type 1 in pregnancy
Source: Endocrine. 2021 May 27;74(1):5–10. doi: 10.1007/s12020-021-02763-5 (PMC8440273; doi:10.1007/s12020-021-02763-5)
Supplement: Supplementary file 1 — Supplementary Information [file 12020_2021_2763_MOESM1_ESM.docx]

**Supplementary**

Figure S1: PRISMA strategy applied for the research of best anti-hypertensive treatment of FH-1 patients during pregnancy.


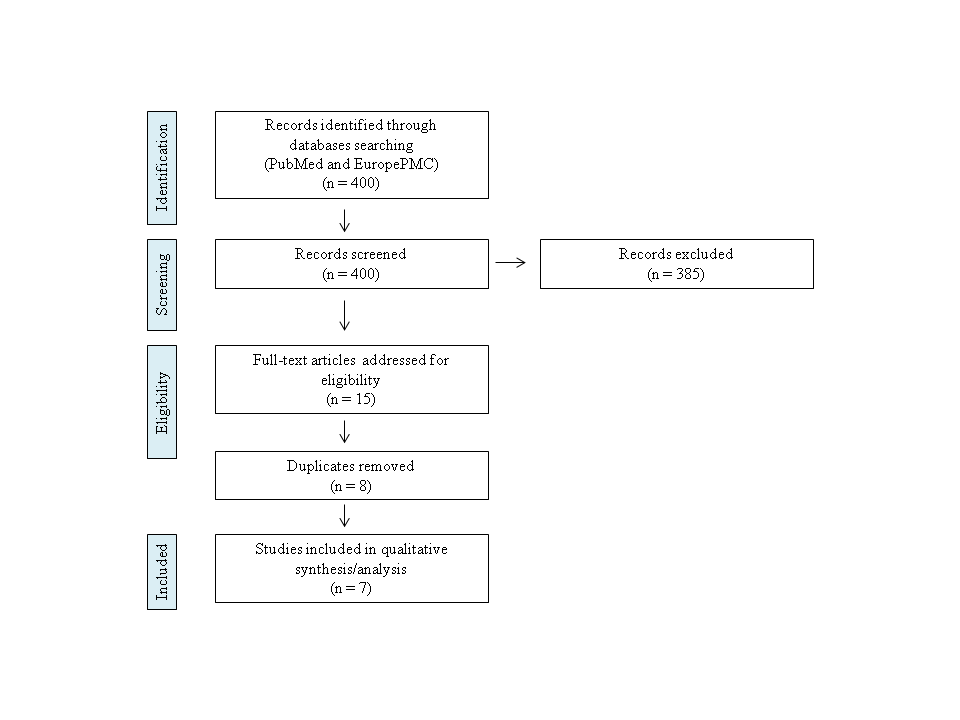


Table S1: PICO strategy applied for the research of best anti-hypertensive treatment of FH-1 patients during pregnancy.

|  | **Description** |
| --- | --- |
| **P** | Familial hyperaldosteronism type 1 OR Glucocorticoid remediable aldosteronism |
| **I** | Treatment in pregnancy OR pregnant women |
| **C** | Usual anti-hypertensive drugs OR glucocorticoid treatment |
| **O** | Blood pressure OR delivery OR foetal outcomes OR newborn outcomes OR maternal outcome OR ceasaran section |

Table S2: Clinical and hormonal data for each trimester in published cases of FH-1 in pregnancy.

| **Reference** | | **Wyckoff**  **et al.** (18) | **Mulatero**  **et al.** (19) | **Hamilton**  **et al.** (17) | **Campino**  **et al.** (16) | **Sanga**  **et al.** (20) |
| --- | --- | --- | --- | --- | --- | --- |
| **Therapy** | **before pregnancy** | N/A | N/A | verapamil | dexa 0.25 mg | dexa 0.50 mg |
|  | **1^st^ trimester** | 17% were treated with undefined drugs | N/A | discontinued verapamil once pregnancy was known | discontinued dexa. 0.25 mg once pregnancy was known | discontinued dexa. 0.50 mg once pregnancy was known |
|  | **2^nd^ trimester** | N/A | N/A | none | none | restarted dexa 0.25 mg at the end of 2^nd^ trimester |
|  | **3^rd^ trimester** | 23% required ≥1  anti-hypertensive medications:  methyldopa (n=2)  potassium-sparing diuretics (n=3)  beta-blockers (n=2)  thiazides (n=5) | N/A | none | none | dexa 0.25 mg |
|  | **after pregnancy** | N/A | N/A | commenced prednisolone 2.5 mg at 1 month postpartum | N/A | dexa 0.50 mg |
| **Blood pressure**  **(mmHg)** | **before pregnancy** | 134±23 / 87±21 | N/A | 200/100 | 110/70 | 123/80 |
|  | **1^st^ trimester** | N/A | N/A | nv | nv | nv |
|  | **2^nd^ trimester** | N/A | N/A | nv | 103/69 | N/A |
|  | **3^rd^ trimester** | N/A | N/A | nv | 110/70 | nv |
|  | **after pregnancy** | 132±16 / 81±11 | N/A | 160/120 | 136/94 | 120/80 |
| **PAC**  **(pmol/l - ng/dl)** | **before pregnancy** | N/A | N/A | 1121 pmol/l  (40.4 ng/dl) | 433 pmol/l  (15.6 ng/dl) | 258 pmol/l  (9.3 ng/dl) |
|  | **1^st^ trimester** | N/A | N/A | N/A | N/A | N/A |
|  | **2^nd^ trimester** | N/A | N/A | At week 18^th^:  260 pmol/l  (9.4 ng/dl)  At week 24^th^:  685 pmol/l  (24.7 ng/dl) | 1772 pmol/l  (63.9 ng/dl) | N/A |
|  | **3^rd^ trimester** | N/A | N/A | N/A | 1223 pmol/l  (44.1 ng/dl) | N/A |
|  | **after pregnancy** | N/A | N/A | 605 pmol/l  (21.8 ng/dl) | 1093 pmol/l  (39.4 ng/dl) | 61 pmol/l  (2.2 ng/dl) |
| **PRA**  **(ng/ml/h)** | **before pregnancy** | N/A | N/A | < 0.10 | 0.9 | 0.1 |
|  | **1^st^ trimester** | N/A | N/A | N/A | N/A | N/A |
|  | **2^nd^ trimester** | N/A | N/A | 2.7 week 18^th^  7.0 week 24^th^ | 4.6 | N/A |
|  | **3^rd^ trimester** | N/A | N/A | N/A | 1.6 | N/A |
|  | **after pregnancy** | N/A | N/A | 0.29 | < 0.2 | 0.9 |
| **ARR**  **(ng/mIU)** | **before pregnancy** | N/A | N/A | 490 | 20 | 113.4 |
|  | **1^st^ trimester** | N/A | N/A | N/A | N/A | N/A |
|  | **2^nd^ trimester** | N/A | N/A | 3.5 week 18^th^  3.5 week 24^th^ | 20 | N/A |
|  | **3^rd^ trimester** | N/A | N/A | N/A | 30 | N/A |
|  | **after pregnancy** | N/A | N/A | 90 | 240 | 3.0 |
| **s-K^+^**  **(mmol/l)** | **before pregnancy** | N/A | N/A | N/A | 3.9 | 3.4 |
|  | **1^st^ trimester** | N/A | N/A | N/A | N/A | nv |
|  | **2^nd^ trimester** | N/A | N/A | N/A | 4.3 | nv |
|  | **3^rd^ trimester** | N/A | N/A | N/A | 4.0 | nv |
|  | **after pregnancy** | N/A | N/A | N/A | 4.2 | 3.5 |

Abbreviations: nv: normal values; N/A: not available; PRA: plasma renin activity; PAC: plasma aldosterone concentration.
